# Polychlorinated Biphenyls (PCBs) and Sex Hormone Concentrations in Ringed and Grey Seals: A Possible Link to Endocrine Disruption?

**DOI:** 10.1007/s00244-020-00716-z

**Published:** 2020-02-27

**Authors:** G. M. Troisi, S. J. Barton, O. Liori, M. Nyman

**Affiliations:** 1 Department of Mechanical & Aerospace Engineering and Institute for Environment, Health & Societies, College of Engineering, Design and Physical Sciences, Brunel University London, Kingston Lane, Uxbridge, UB8 3PH UK; 2 Department of Chemical and Pharmaceutical Sciences, Kingston University, Penrhyn Road, Kingston-upon-Thames, Surrey KT1 2EE UK; 3 Finnish Game and Fisheries Research Institute, Metsähallitus, Kirjaamo, P.O. Box 94, Vantaainland, 01301 Finland

## Abstract

Polychlorinated biphenyls (PCBs) are recognised reproductive and immune system toxicants in marine mammals mediated by endocrine-disrupting mechanisms. As with other predators, seals are exposed to elevated bioaccumulated concentrations of PCBs and other persistent organic pollutants (POPs). Cryopreserved plasma samples from adult ringed (*Phoca hispida; n* = 39) and grey (*Halichoerus grypus; n* = 38) seals, sampled between 1998 and 2002 from Baltic Sea, Svalbard, and Sable Island (Canada) were used to investigate relationships between PCB exposure and sex hormone concentrations (progesterone; P4, 17α-hydroxy progesterone; 17α-OH-P4, testosterone; T4, 17β-estradiol; E2, estrone; E3). Immunoassay methods were used for quantification of analytes due to the limited sample volumes available. PCB concentrations were found to be significantly higher in Baltic seals than other sampling locations and were classed as “*Exposed”* seals while Svalbard and Sable Is seal were classed “*Reference”* seals (sexes and species separate). Mean hormone concentrations in *Exposed* seal were lower than *Reference* seals, and this was statistically significantly for 17α-OH-P4 (both sexes and both species), E2 (ringed and grey seal females), and E3 (grey seal females). Regression analyses (PCB v hormone concentrations) for each sex and species revealed significant correlations for P4 (Sable Is. female grey seals and female ringed seals), 17α-OH-P4 (Sable Is. male grey seals and Svalbard male ringed seals), T4 (Svalbard male ringed seals), E2 (female ringed seals), and E3 (female ringed seals and Baltic female grey seals). Although significant correlations are not evidence of cause and effect, the potential impact of hormone changes on endocrine homeostasis and reproductive health for seal populations warrants further investigation given that PCB concentrations found here are in the same range as those currently reported in seals from these populations.

Being a heavily industrialised area with many sources of industrial pollution, the Baltic marine food chain continues to be contaminated with heavy metals, polyaromatic hydrocarbons, and persistent organic pollutants (POPs). Polychlorinated biphenyls (PCBs) and organochlorine pesticides, such as dichloro-diphenyl-trichlorethane (DDT), polybrominated flame retardants (polybrominated diphenyl ethers [PBDEs] and polybrominated diphenyls [PBBs]) are apolar, lipophilic, persistent, and ubiquitous environmental pollutants. As apex predators, marine mammals are exposed to biomagnified concentrations of these POPs via their diet, accumulating highest levels in lipid-rich tissues, such as blubber (Brown et al. 2018). Exposure to organochlorines cause a variety of adverse health effects, including hepatic, reproductive, and immune toxic effects often involving endocrine-disrupting toxic mechanisms. Endocrine-disrupting chemicals (EDCs) are defined as “exogenous chemicals, capable of interfering with normal endocrine homeostasis, such as the production, release, metabolism, binding, action, and elimination of endogenous hormones (Colborn et al. 1993). Reproduction is intrinsically controlled by endocrine signals in response to maturation and environmental cues via the hypothalamus–pituitary–gonadal axis. The capacity of a seal’s homeostatic mechanisms to rectify the influence of endocrine disruption may be exceeded where significant organohalogen pollutant exposure exists. This is especially true at sensitive stages, such as prenatal and perinatal development, puberty, and gestation. Studies have shown that organochlorine exposure disturbs normal hepatic steroid metabolism and circulating sex and thyroid hormone concentrations, resulting in reduced fertility, immunosuppression, and reproductive failure in marine mammals (DeLong et al. 1973; Reijnders 1986; Brouwer et al. 1989; deSwart et al. 1996; Haave et al. 2003; Ropstad et al. 2006; Routti et al. 2010; Villanger et al. 2013; Gustavson et al. 2015; Ciesielski et al. 2017). Some of the most highly contaminated animals historically have been the Baltic seals and population-level impacts from organochlorine exposure have occurred. For example, PCBs and DDTs were believed to have induced uterine deformities and sterility in Baltic Seals, causing population size to plummet from ~ 14,000 in the 1960s to 4000 in the early 1980s (Helle et al. 1976a, b; Helle 1980; Harding et al. 2007; Harding and Härkönen 1999). Also, PCBs and DDT have immunosuppressive effects in seals (deSwart et al. 1996).

Since the enforcement of international bans on POPs (UNEP 2017), concentrations have declined in Northern European, Canadian Atlantic, and Arctic marine ecosystems, and this is mirrored in tissue concentrations of local seal populations (Muir and deWit 2010; Ross et al. 2013; Bjurlid et al. 2018; Brown et al. 2018; HELCOM 2018a, b). However, these “legacy chemicals,” due to their persistent nature and global distribution, still persist and PCBs remain the greatest contribution to total organic pollutant burdens in seals and other apex predators (Greaves et al. 2012; Shaw et al. 2012; Brown et al. 2018; AMAP 2018; Schnitzlera et al. 2019; Robinson et al. 2019). In this study, the effects of PCBs on circulating sex hormones (progesterone [P4]; 17α-hydroxy progesterone [17α-OH-P4]; testosterone [T4]; 17β-estradiol [E2]; estrone [E3]) were investigated as evidence for endocrine disruption in wild seal populations. Suitably cryopreserved blood samples from ringed (*Phoca hispida*) and grey (*Halichoerus grypus*) seals (sampled between 1997 and 2002) that were surplus to a previous study were used. Blood was chosen as the sample medium, because it reflects physiologically relevant exposure in real time that is circulating to target organs/tissues and interfering with hormone transport. Because there is good evidence historically that Baltic seals are of the most contaminated populations with POPs, these were identified as “*Exposed*” seals, whose PCB and hormone concentrations could be compared with those of less exposed “*Reference*” seals known to have lower POPs concentrations from Svalbard (Arctic) and Sable Island (Canada) populations (Nyman et al. 2002; Routti et al. 2008, 2010; Shaw et al. 2012). Second, changes in sex hormones over a gradient of environmental PCB exposures also were investigated by dose–response analyses for each gender and species.

## Methodology

### Sampling and Study Populations

It should be noted that seals were not sacrificed for the purposes of this study. Samples used were surplus samples remaining from a previous study by Nyman and co-workers of the *Finnish Game and Fisheries Research Institute* (Finland). Nyman and co-workers sampled seals from Baltic (Bothnian Bay) and Svalbard seal populations under local hunting law with special permission granted from the *Ministry of Forestry and Agriculture* (Finland) to Nyman and co-workers for their research project (Nyman et al. 2002). Sable Island (Canada) samples were collected with special permission granted to the host sampling institute in Canada (*Marine Institute by the Bedford Institute of Oceanography*). Two species were sampled as follows: Ringed seals (*Phoca hispida*) from the Baltic Sea and Svalbard and Grey seals (*Halichoerus grypus*) from the Baltic Sea and Sable Island populations. Adult seals were sampled from both sexes at random with an average age of 12.6 years (range 5–33; age and sex distributions were not significantly different between populations; Nyman et al. 2002). The seals were sampled after the mating season during their respective annual moult: April–May in the Baltic Sea and May–June in Sable Is. and Svalbard between 1997 and 2002. At the time of sampling, sexually mature females were either gestating at the stage of embryonic diapause (or pseudo-pregnant) or actively gestating (only Sable Is. grey seals), whereas males were quiescent following the mating season. Preparation of samples was described by Nyman et al. (2002) as follows; samples were transported on dry ice to the lab to maintain sample quality, and there whole blood was centrifuged at 3000 rpm for 5 min to prepare the plasma which was stored at − 70 °C until analysis. For this study 77 samples from ringed and grey seals were obtained and analysed for PC and sex hormones in 2004. Samples sizes were unequal from each species and populations, due to limited samples from the archive. Steroid hormones in mammalian serum/plasma have been shown to be stable at − 70 °C by several studies, as reviewed by Biery (2013); the longest storage duration studied was 30 years (Kley and Rick 1984; Cauley et al. 1987; Bolleli et al. 1995; Stroud et al. 2007; Zhang et al. 2007; Holl et al. 2008). Due to the limited sample volumes available, immunoassay methods were used for quantification of PCBs and steroid hormones, which require microvolume samples and chromatographic methods used for a subsample where larger volumes were available for ELISA method validation.

### Chemicals

Reagents used for 17β-estradiol (E2), estriol (E3), progesterone (P4), 17-hydroxy progesterone (17α-OH-P4), and testosterone (T2) assays were all supplied in kit form by *DRG*^®^*Diagnostics* (DRG International Inc., USA). Aroclor 1254 standard (technical polychlorinated biphenyl (PCB) congener mixture), chromogen (3,3′,5,5′-tetramethyl benzidine (TMB) with hydrogen peroxidase), pentachloro-phenol (PCP)-horseradish peroxidase (HRP), and polyclonal rabbit anti-PCB antibody (raised against Aroclor 1254) were supplied in kit form from *Abraxis LCC* (USA). All solvents used were glass distilled grade and obtained from *Rathburn Chemicals Ltd* (Scotland). Bovine serum albumin (BSA), and all other reagents were supplied by *Sigma*-*Aldrich Inc.* (USA). All reagents were stored at 4 °C and used within 24 h of preparation. In preparation for analysis, serum samples were equilibrated to 5 °C following a short thawing period from − 70°C and gently mixed to ensure homogeneity.

### Hormone Quantification

Sample volumes were limited and varying in volume such that not all analytes could be analysed in every sample, resulting in unequal sample sizes for each analyte tested. E3 was not quantified in samples from males as this hormone occurs in pregnancy, formed by placental metabolism of fetal 16α hydroxy-dehydroepiandrosterone sulphate (DHEA-S) and increases with gestation, making it a valuable hormone for diagnosing fetal distress and progression of gestation (Carr 1996). Due to limited sample numbers, T4 could not be analysed in female grey seals and P4 in male grey seals. Otherwise, all hormones were quantified for all seals. Hormone concentrations were determined by commercially available solid phase enzyme-linked immunosorbent assay enzyme-linked immunosorbent assay (ELISA) kits using polyclonal antibodies to E2, E3, P4, 17α-OH-P4, and T2. High specificity of the capture antigens of the commercial kits was determined from the manufacturer’s cross-reactivity data (*DRG*^*®*^*Diagnostics, DRG International Inc., USA*). The assays were based on a competitive binding principle where any hormone present in the samples competes with horseradish peroxidase (HRP)-conjugated (labelled) hormone for binding sites on the capture antibody, which had been precoated on to the microplates. Following an incubation period, unbound HRP conjugate was removed. HRP substrate solution was added to generate a colour whose intensity is inversely proportional to the concentration of the hormone in the sample/standard. Kits were used according to the manufacturers’ instructions. Briefly, 25-µl aliquots of plasma sample (or standard or blank as appropriate) were added to each well. After a 5-min incubation period at room temperature, 200 µl of HRP conjugate were added to each well and gently aspirated. Plates were incubated at room temperature for 60 min, then washed with washing solution, and the washing gently removed. In each well, 200 µl of TMB substrate were added. After 15 min, reactions were stopped by adding 100 µl of 0.5 M H_2_SO_4_ to each well. Absorbance was measured at 450 nm using a microplate plate reader (*Bio*-*Rad Laboratories*). Samples, blanks, and standards were analysed in triplicate. Concentrations of analytes were extrapolated from the linear portion of the respective standard curves for each analyte. Recovery was determined for each analyte with spiked samples included with every test batch. Sample concentrations were adjusted accordingly for losses determined for each batch. ELISA validation data are summarised in Table 1. Briefly, parallelism was investigated by linear regressions of values for serial dilutions of spiked seal plasma with high hormone concentration and corresponding standard curve for each hormone (*r*^2^ > 0.98 in all cases), indicating minimal matrix effects. Linearity was tested for all hormones over ranges of sample concentrations and standard curves for each hormone.

**Table 1:** Assay validation data for hormone ELISA kits

Analyte	Intra-assay mean CV ± SD (%)	Inter-assay mean CV ± SD (%)	Recovery mean ± SD (%)	Parallelism (*r*^2^)	Linearity (*r*^2^)
E2	7.3	5.9	113.3 ± 7.1	0.981 (*p* < 0.005)	0.995 (*p* < 0.001)
E3	8.1	6.3	91.9 ± 5.2	0.979 (*p* < 0.005)	0.998 (*p* < 0.001)
T4	6.8	11.8	91.4 ± 6.7	0.973 (*p* < 0.005)	0.997 (*p* < 0.005)
P4	4.9	7.2	94.1 ± 2.4	0.977 (*p* < 0.001)	0.997 (*p* < 0.001)
17-OH P4	6.2	10.8	108.7 ± 9.1	0.988 (*p* < 0.005)	0.992 (*p* < 0.001)

*CV* coefficient of variation (mean %); *SD* standard deviation

### PCB Quantification

A commercially available competitive ELISA kit was used to quantify PCBs in serum samples (PCB ELISA Micro-titre Plate kit, *Abraxis LLC*, USA). The ELISA was conducted according to the manufacturer’s protocol with minor modification to optimise sensitivity and control interference from nonspecific binding (matrix effects) from the serum matrix. Before analysis, serum samples were mixed with diluent solution (50:50 v/v methanol/tris–HCl buffered saline, 150 mM NaCl, 7% BSA, pH 7.6). PCB calibration standard (A1254) was diluted with the same diluent solution to achieve a working concentration range of 0–250 ng/ml (ppb). Working dilutions of PCB antibody was 1:5000 and PCB-HRP conjugate was 1:2500, diluted with 50 mM Tris–HCl buffered saline, 0.1% BSA (pH 7.4). These dilutions were found to generate optimal absorbance range/colour reaction for good assay sensitivity. Microtiter well strips were provided by the manufacturer precoated with rabbit anti-PCB antibody. For the assay, each well was loaded with 50 µl of sample or standard, 50 µl of PCP-HRP, and 50 µl of PCB antibody. The plate was incubated for 1 h at room temperature. Following this, plates were washed three times with 250 µl of washing buffer (50 mM Tris–HCl, 0.05% Tween 20, pH 8.0). Then, 150 µl of enzyme–substrate chromogen (TMB in H_2_O_2_) were added to wells. Reaction was terminated by the addition of 100 µl of 2 N H_2_SO_4_ to each well after 20 min. Absorbance was measured at 450 nm using a microplate reader (*Bio*-*Rad Laboratories Inc.*).

PCB concentrations in samples were extrapolated from calibration curves and the limit of detection (assay sensitivity) was 0.03 µg/ml. To minimise interplate variation, samples were analysed in triplicate on the same plate, and PCB concentrations were extrapolated from the calibration curve derived on the same plate. A blank (duplicate) also was analysed with each sample set to control any background absorbance attributable to nonspecific binding and to avoid overpresentation of the data. Within assay precision (mean coefficient of variation (% CV = 6.8 ± 1.8%), interassay reproducibility (% CV = 12.3 ± 2.7%) and PCB recovery (range 80–105%) were all within acceptable limits. To validate the ELISA method used in this study, PCB concentrations data were compared with data obtained from the analysis of replicate blood samples determined by traditional gas chromatographic-mass spectroscopy (GC–MS) methods. Full details of sample preparation and analytical procedures are described by Nyman et al. (2002). Linear regression analysis showed significant correlation between ELISA and GC–MS determined PCB concentrations (*r*^2^, *p *< 0.922; *n* = 26).

## Results and Discussion

### PCB Concentrations

The range of PCB concentrations found in ringed (range 0.88–46.87 µg/ml) and grey seals (range 0.30–87.06 µg/ml) corresponded with ranges reported by others for these seal populations (Bang et al. 2001; Nyman et al. 2002; Routti et al. 2008, 2010; Bjurlid et al. 2018). Influence of sex, sampling location, and species on PCB concentrations in seal groups were investigated by ANOVA with sex, species, and sampling location (Svalbard/Sable Island or Baltic) assuming 5% significance. Pairwise comparison across species and gender showed PCB concentrations were significantly higher in Baltic than Svalbard seals (*p* < 0.0001). Also, across sampling location and gender, PCBs were significantly higher in grey than ringed seals (*p* = 0.024). Across both species and both regions, males (ringed 11.72 ± 11.21 µg/ml and grey 26.26 ± 28.10 µg/ml) had significantly higher concentrations than females (ringed 7.98 ± 6.76 µg/ml and grey 7.62 ± 11.3 µg/ml) (*p* = 0.004). This was an expected finding since PCB accumulation is sex-dependent in marine mammals (females eliminate POPs via lactation and parturition and levels increase with age in males; Peterson et al. 2014) and this also was reported for the same seals by Nyman et al. (2002). ANOVA revealed a statistically significant *interaction effect* between gender and species. Specifically, for both sampling locations (Baltic and Svalbard/Sable Is), the difference in PCB levels between genders increased between ringed and grey seals. For Svalbard/Sable Is. sampling locations, PCB concentrations in both genders were similar in ringed seals, but the difference increased (significantly) for grey seals, with males having higher concentrations than females. Lastly, PCB concentrations for grey seal females from Sable Is. were lower than those for female ringed seals from Svalbard. The situation was reversed for the Baltic. PCB levels in females were slightly higher than in those in Svalbard/Sable Is. For Baltic males, PCB levels increased between ringed and grey species. It should be noted that unavoidable error was introduced in our tests by using ANOVA because of our unequal sample sizes. It was necessary to randomly remove values from larger sample groups to equalise sample sizes to facilitate ANOVA testing. A more detailed study with larger sample sizes, influence of age, body condition, location, and sex on the variation in PCB burdens in the seals of this study are described by Nyman et al. (2002). It is well established that PCB concentrations in different seal species are influenced by several factors, such as differences in hepatic CYP450-dependent PCB detoxification potential, where ringed seals have a lower capacity for metabolising planar PCB congeners than grey seals (Nyman et al. 2001). Also, differences in foraging behaviour between species influence variation in PCB concentrations. For example, Baltic grey seals seasonally feed in southerly areas and the Bothnian Bay, whereas Baltic ringed seals, being more sedentary, forage mainly in the Bothnian Bay throughout the year (Nyman et al. 2002). The threshold concentration beyond which adverse reproductive effects are expected to occur in seals is 25–77 µg/g (lipid weight) PCB in blubber (Helle et al. 1976b; AMAP 2000). Although the threshold is given for blubber concentrations, blood PCB concentrations detected in some of the Baltic Seals in this study, fell within this range and so may have been “at risk” of adverse reproductive effects.

**Fig. 1:**
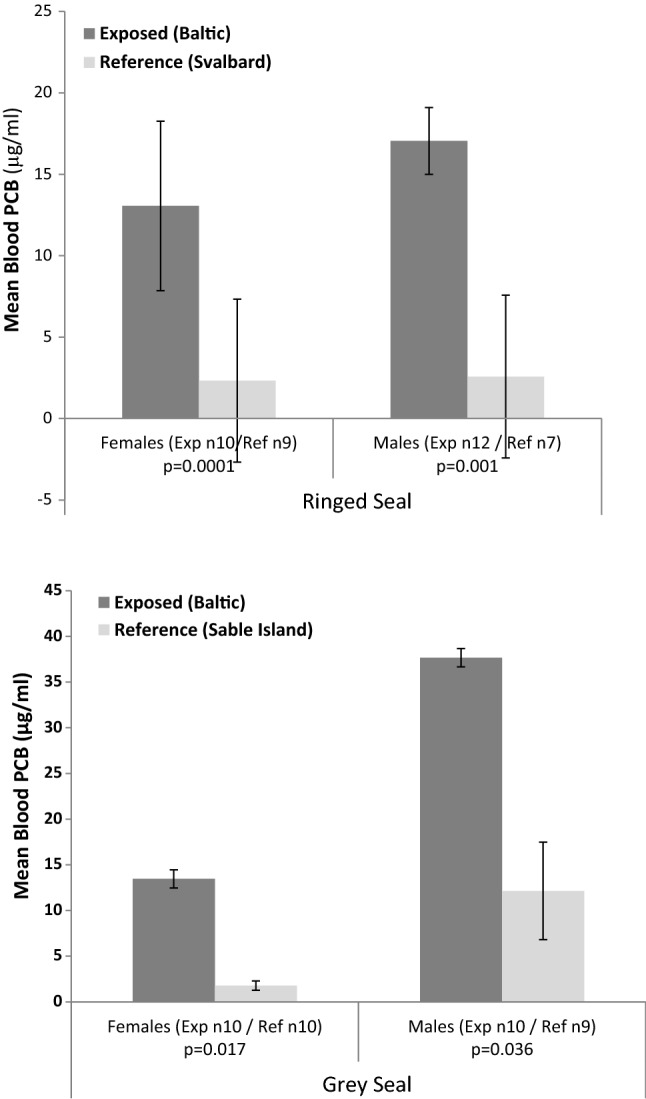
Mean PCB (mean ± SD) concentrations (wet weight) in “*Exposed*” and “*Reference*” seal groups

To categorise seals as “*Reference”* (low concentrations of PCBs) or “*Exposed”* (high concentrations of PCBs), we compared mean PCB concentrations on the basis of sampling location, gender, and species. Mean PCB ± standard deviations concentrations for each group, and seal groups are presented in Fig. 1. Student *t* tests (assuming equal or unequal variance, depending on *F*-test for two-sample variance, assuming 5% significance) were used to determine the significance of differences over ANOVA to avoid the error from deleting values to equal samples sizes in already small sample groups. To minimise confounding factors on PCB concentration, four groups were created for each species (*Reference* Females; *Reference* Males; *Exposed* Females; *Exposed* Males). For ringed seals, the Null Hypothesis “*No significant difference in mean PCB concentration in Baltic and Svalbard seals”* was rejected, as Baltic seals had significantly higher mean PCB concentrations so ringed seals could be classed as “Exposed” seals and Svalbard ringed seals as “Reference” seals (true for both sexes). For grey seals, the Null Hypothesis “*No significant difference in mean PCB concentrations in Baltic and Sable Is. seals”* also was rejected. Again, Baltic grey seals could be classed as “Exposed” grey seals, whereas Sable Is. seals could be classed as “Reference” seals (true for both sexes).

### Hormone Concentrations

**Fig. 2:**
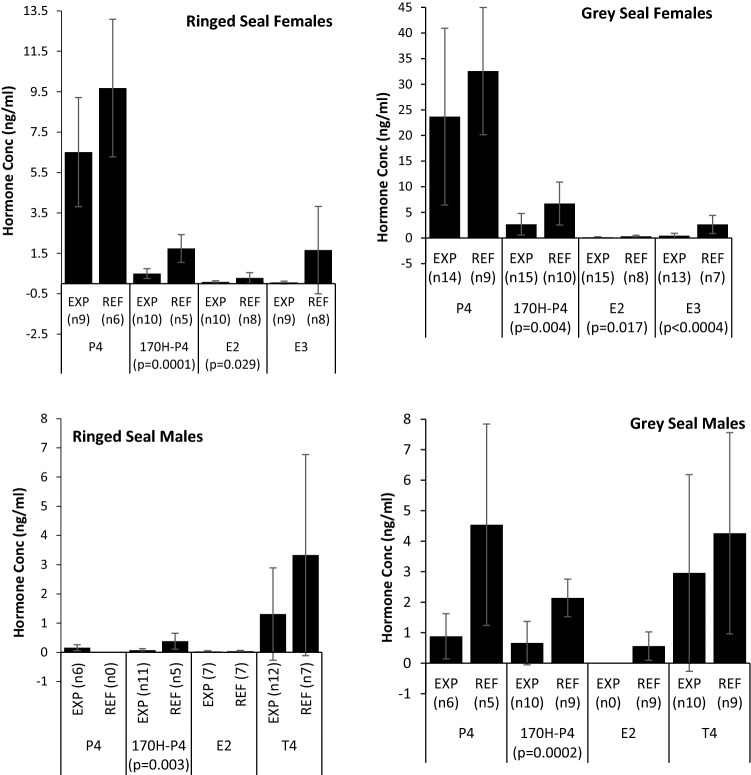
Mean hormone (mean ± SD) concentrations in “*Exposed*” and “*Reference*” seal groups

Mean hormone concentrations in are presented in Fig. 2. This is the first study of plasma sex hormone concentrations in ringed seals, with the exception, of T4 studied previously in males, and for grey seals, the first report of E3 and 17α-OH-P4. In mammals, P4 is synthesised by CYP17α hydroxylation of pregnenolone and in turn the same isozyme converts P4 to its primary metabolite 17αOH-P4 (Bremer and Miller 2014). Detection of 17αOH-P4 in plasma samples in this study indicate ringed and grey seals have the capacity for CYP17A1-mediated 17α hydroxylation activity. Similarly, detection of T4 and E2 in blood samples indicates gonadal isozymes of ringed and grey seals exhibit CYP19A1 aromatase activity, which is the isozyme converting T4 to E2 in mammals (Bremer and Miller 2014). More specifically, in vitro studies with grey seals liver microsomes showed that 2β (CYP3A), 6β (CYP3A, CYP1A), and 16β (CYP2B) hydroxylation of T4 in this species (Li et al. 2003). P4 and E2 levels in female ringed and grey seals were in the same range as those reported for harbour, grey seals, and spotted seals at the same reproductive stage (Boyd 1983; Reijnders 1990; Gardiner et al. 1996; Mellish and Iverson 2005; Lydersen and Kovacs 2004; Zhang et al. 2014). For both species studied, T4 concentrations were higher than those reported in the only other report of T4 in female pinnipeds in otariid seals (Browne et al. 2006). Because 17α-OH-P4 levels have not been reported before in female pinnipeds, we compared with female Sledge Dogs, and they were in the same range (Sonne et al. 2014). For males of both species, we found T4 concentrations were similar to those published for male ringed, harbour, grey, and spotted seals (Seely and Ronald 1991, Coltman et al. 1999; Lydersen and Kovacs 2004, Krafft et al. 2007; Zhang et al. 2014). Mean P4 and E2 concentrations in males of both species studied were similar to those reported in male spotted seals (Zhang et al. 2014). The sex-dependent trends observed here are well established for pinnipeds and other marine mammals (Pomeroy 2011).

We used Student’s *t* tests (assuming equal or unequal variance, depending on *F*-test for two-sample variance, assuming 5% significance) to determine the significance of any differences in mean sex hormone concentrations between seal groups. ANOVA was not used to prevent introducing error by reducing our already limited data sets to generate equal sample sizes required for this type of test. Mean hormone concentrations in *Reference* and *Exposed* seals for each species are presented (sexes separate) in Fig. 2. T4 concentrations were significantly higher in males than females for each species (grey seals; *p* < 0.0001 and ringed seals; *p* < 0.06), whereas P4, 17α-OH-P4, E2, and E3 were higher in females than males for each species but statistically significant for P4 (*p *< 0.0001), 17-OH-P4 (*p *= 0.001), E2 (*p *= 0.02), E3 (*p *= 0.02) in grey seals and 17α-OH-P4 (*p *= 0.002) and E2 (*p *= 0.03) in ringed seals. For all hormones, mean concentrations were lower in *Exposed* than *Reference* seals, and these differences were tested by Student’s *t* test as follows. For male ringed seals, the Null Hypothesis, “*No significant differences in mean concentration between Baltic and Svalbard seals”* was rejected for 17α-OH-P4 (*p *< 0.003) and for females was rejected for 17α-OH-P4 (*p* < 0.0001) and E2 (*p* < 0.029). P4 in males was not compared due to insufficient samples. In grey seals, mean hormone concentrations were again lower in *Exposed* than *Reference* seals. The Null Hypothesis, “*No significant differences in mean hormone concentration between Baltic and Sable Island seals*” was rejected for females for 17α-OH-P4 (*p* < 0.004), E2 (*p* < 0.017), and E3 (*p* < 0.0004) and for 17α-OH-P4 in males (*p *< 0.0002). T4 in males was not due to insufficient samples. Although it is difficult to draw comparisons with our data, lowered sex hormone concentrations also were reported in the exposed groups of controlled PCB dosing studies with sledge dogs, harbour seals, mink, and Arctic foxes (Reijnders 1986; Shipp et al. 1998; Hallanger et al. 2012; Sonne et al. 2014). To our knowledge, there are no studies of E3 in seals to compare our data, and this is the first evidence of any link between PCB exposure and E3 concentrations in aquatic mammals. Maternal circulating E3 concentration is used a marker of fetal health in human medicine (Carr 1996). Physiologically, normal E3 concentrations over the duration of gestation in pinnipeds are not available, so it was not possible to diagnose fetal health from our data. However, if mean E3 values for *Reference* seals are taken as “normal” values, then corresponding E3 levels in *Exposed* seals could possibly indicate poorer fetal health in this group. These findings suggest that PCB exposure could lower circulating hormone concentrations in environmentally exposed seals. As most females in this study were gestating when sampled, any xenobiotic inducted reduction in hormone levels could have deleterious influence on pregnancy outcomes. Clearly, larger sample sizes are needed to verify these interpretations from our very limited sample size.

**Fig. 3:**
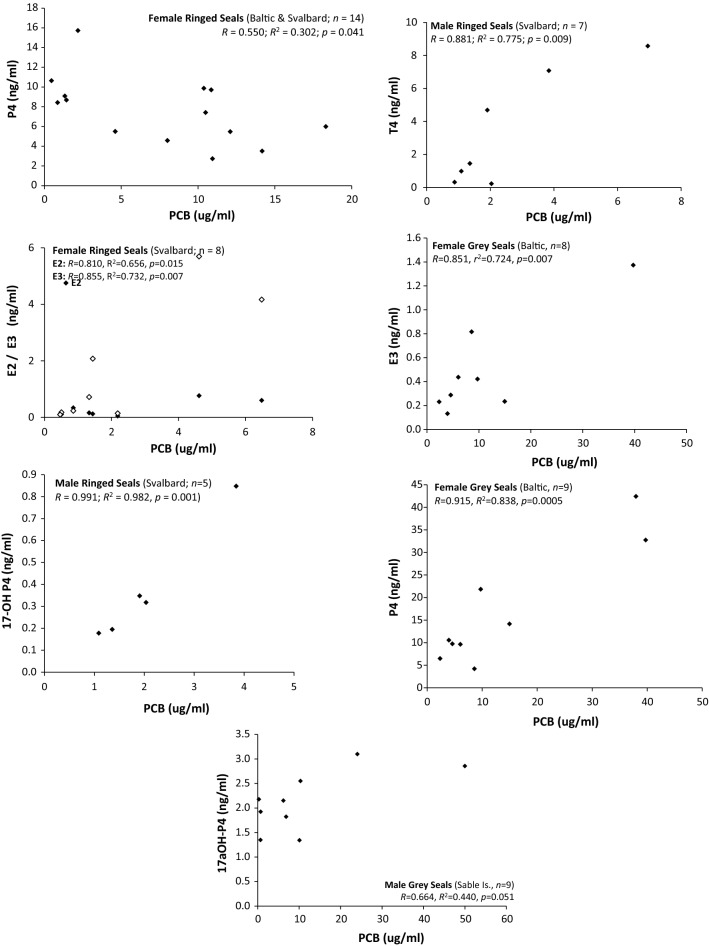
PCB concentration versus sex hormone concentration scatterplots for seal groups (significant correlations only)

We used our data to investigated exposure–response relationships between PCB and hormone over a range of concentrations by regression analysis for each group of seals. Seals from each location were sampled at the same stage of the annual reproductive cycle; therefore, data for seals from each location were combined (species separate) to investigate correlations over a larger range of PCB concentrations. Due to the known sex-dependent differences in hormone concentrations, we considered sexes separately. The hormones for which statistically significant correlations were observed are presented in Fig. 3. A significant positive correlation between PCB and P4 was observed for female grey seals (Baltic and Sable Is. combined) as observed in female polar bears by Haave et al. (2003) and Ropstad et al. (2006). However, in female ringed seals (Baltic and Svalbard combined), a significant negative correlation was observed, contrasting with that seen in female grey seals or that which was reported in female polar bears (Ropstad et al. 2006). Significant positive correlations of PCB v 17α-OH-P4 were observed in female ringed seals (Svalbard) and male grey seals (Sable Is.). Because this is a major P4 metabolite via CYP17-hydroxylation (Sanderson, 2006), this may support the positive correlation observed for PCB v P4 in female grey seals already described. A significant positive correlation was observed between PCB and T4 in Svalbard male ringed seals, which contrasts with negative correlations observed in environmentally exposed male polar bears (Ropstad et al. 2006; Ciesielski et al. 2017) and Dall’s porpoises (Subramanian et al. 1987). However, it has been shown that in male polar bears, circulating T4 concentrations are more influenced by body condition than exposure to POPs (Ropstad et al. 2006; Ciesielski et al. 2017). Significant positive correlations also were observed between PCB and the following estrogenic hormones: E2 and E3 in female ringed seals (Baltic and Svalbard combined), and E3 in Baltic female grey seals. For all other seal groups, correlations between PCB and sex hormones were not statistically significant. It should be noted that the comparisons made between our results and other published studies are confounded by the different analytical methods used to quantify PCBs. Ideally, the combination of immunoassay methods with high resolution congener-specific PCB analysis would offer further insights into the relationships between exposure and hormone changes.

In mammals, PCBs interfere with the biosynthesis, transport, and metabolism of steroids by altering the activity of cytochrome P450 isozymes, such as 5α-reductase, 3α-, 11β-, and 17β-hydroxy steroid dehydrogenases (Sanderson 2006; Romeo et al. 2009; Hampl et al. 2014). In vitro studies of hepatic microsomal hormone metabolism in harbour and grey seals have shown these species have the capacity for 6β- and 2β-hydroxylation of T4 and 6β- and 16α-hydroxylation of P4 indicative of CYP1A, CYP3A, and CYP2B isozyme activity, as occurs in most mammals in biosynthesis (Troisi and Mason 2000; Li et al. 2003). These isozymes also are known to be induced by PCBs in seals, as with other mammals, during phase I hepatic detoxification processes (Boon et al. 1992; Nyman et al. 2001). In harbour seals, both P4 and T4 metabolism were negatively correlated with PCB concentration, indicative of an inhibitory effect of PCBs on hormone metabolism and excretion (Troisi and Mason 2000; Li et al. 2003). Several steroid-metabolising isozymes, such as CYP1A and CYP2B, also are critically involved in the metabolism and detoxification of PCBs (Amacher 2010; Hampl et al. 2014). It is proposed that the reductions in circulating hormone concentrations in more exposed seals in this study, resulted from altered CYP450-dependent steroid biosynthesis and metabolism, caused by PCB induced inhibition of CYP450 isozymes and/or concurrent depletion of isozymes from phase I hepatic microsomal detoxification of PCBs and other organic pollutants. This toxic mechanism also has been proposed by others, following the findings of in vivo PCB dosing students and in vitro studies of microsomal CYP450 activity (Yoshihara et al. 1982; Machala et al. 1998; Amacher 2010). PCBs also may influence hormone metabolism by changing the expression of transcription factors, which signal the induction of CYP450 isozymes, such as Aryl hydrocarbon receptor (AHR), constitutive androstane receptor (CAR), and pregnane X receptor (PXR) (Amacher 2010). An important exacerbating factor that should be considered is the ability of PCBs and their hydroxylated metabolites to bind sex hormone-binding globulin (SHBG) in blood and intracellular hormone receptor binding sites. These actions potentially displace endogenous hormones, disrupting hormone transport to target organs and hormone signalling processes (Jury et al. 2000). This can worsen the impact of the observed reduced circulating sex hormone levels in more exposed seals. In seals, PCBs and OH-PCB can displace thyroid steroid hormones from blood transport proteins and their intracellular cellular receptor proteins, leading to thyroid hormone disruption and immunosuppression in individuals with highest PCB exposures (Brouwer and van den Berg 1986; Brouwer et al. 1989; deSwart et al. 1996; Routti et al. 2010). In newborn babies, unbound SHBG protein concentration was increased, whereas E2 and T4 levels concomitantly decreased, with increasing PCB exposure, highlighting how receptor level changes can influence circulating hormone levels from PCB exposure (Cao et al. 2008; Warembourg et al. 2016). Methyl sulphones (${\text{MeSO}}_{{2^{ - } }}$) are another group of PCB metabolites able to interfere with the signalling P4-dependent actions in the uterus, because they have high binding affinity for seal uterine P4 receptor protein (Troisi et al. 2001). This interference combined with lowered concentrations of circulating E2 and P4 hormones, vital to the maintenance of pregnancy, could have been important precursory events to the development of uterine occlusions (ringed seals) or stenoses (Grey seals) and infertility in Baltic seals with high PCB concentrations in the 1970s (Olsson et al. 1974; Helle et al. 1976a, b; Helle 1980; Baker 1989). The role of PCBs in reproductive toxicity is well established from controlled feeding studies of harbour seals, mink, and sledge bitches fed PCB-contaminated diet at environmentally relevant concentrations. The authors reported PCB binding with E2 and P4 receptors and concomitant changes in circulating hormone levels leading to embryo resorption, anovulation, fetal resorption, delayed ovulation, increased gestation time, and reduced litter size (Reijnders 1986; Shipp et al. 1998; Sonne et al. 2014).

With the decline of POP pollution in the Baltic Food chain since bans were enforced and the concomitant decline in POPs levels in seals, a positive trend in gynaecological health of Baltic seals has been reported, including a decreased incidence of uterine deformities, uterine leiomyomas, and increased pregnancies (Bergman 1999; Helle et al. 2007; Bäcklin et al. 2013). The current population growth rate is now 5% a year according to the latest Baltic Seal census (Luke 2016). Although the concentrations of PCBs have declined in seals, they remain the predominating contribution to organic chemicals levels in pinnipeds (Bjurlid et al. 2018; Ross et al. 2009; Robinson et al. 2019). PBDEs, with similar modes of action to PCBs, still enter marine food chains as bans of these endocrine-disrupting chemicals were more recent. Some PBDEs are still in use (e.g., pre-ban buildings, furniture and electronic products) and PBDES enter the environment via waste streams (UNEP, 2017). Although only PCB concentrations were measured in this study, synergistic and/or antagonistic effects mediated by the mixtures of endocrine disrupting chemicals contaminating seals were not investigated but are of toxicological importance and warrant further study. Similarly, the influence of diet, body condition, age, specific stage of the reproductive cycle, and other physico-kinetic aspects of individual seals are further confounding factors that influence the findings of this study.

Blood sampling offers ecotoxicologists a nondestructive, more ethical approach to researching wild populations than sacrificing animals, but a better understanding of the toxicological relevance of blood measures for tissue and whole animal effects is needed. Organohalogen quantification by chromatographic methods (e.g., high-resolution GC–MS) is resource-intensive (e.g., time, cost, technical expertise), requiring larger sample volumes for blood analysis. This can disincentivise researchers where large sample sizes are needed for statistical power (e.g., for biomonitoring) and/or where funding is limited. We show that immunoassays can effectively quantify plasma PCB and hormone concentrations in small sample volumes where chromatographic methods are not favourable. Although an introductory approach, it is a starting point towards achieving high throughput, cost-effective pollutant and hormone analyses for wildlife monitoring and should be further developed considering the benefits described.

## Conclusions

Although the findings in this study are not conclusive of cause and effect, they contribute to the significant body of evidence that organohalogen pollutants cause endocrine disruption in seals, from the subcellular level (hormone receptor interactions and altered hormone biosynthesis and metabolism (Troisi and Mason 2000; Troisi et al. 2001; Li et al. 2003; Routti et al. 2010), tissue-organ level (uterine deformities; Olsson et al. 1974; Helle 1980; Baker 1989), individual level (reproductive failure; Reijnders 1986), and ultimately at the population level (localised extinction of Baltic Sea ringed and Grey seals; Helle 1980; Helle et al. 1990; Harding and Härkönen 1999). Although the mode-of-action whereby PCBs disturb hormone homeostasis is unclear, it is likely that disruption of steroid biosynthetic pathways, and the interference with hormone binding to plasma transport proteins and hormone receptor proteins are involved (Sanderson 2006; Romeo et al. 2009; Hampl et al. 2014). Despite international bans of POPs, PCBs continue to contaminate apex predators, and their levels in Baltic and Svalbard seals remain in the same range as those reported in this study (Bjurlid et al. 2018). These seals also are impacted by other anthropogenic factors, including climate change, habitat destruction, hunting, net entanglement, overfishing, epizootics, oil spills, and toxic algal blooms. The continued exposure of aquatic mammals to organohalogen pollutants should be viewed in context of these other threats due to the impact of endocrine disruption on population recovery.
